# A high salivary calcium concentration is a protective factor for caries development during orthodontic treatment

**DOI:** 10.4317/jced.56331

**Published:** 2020-03-01

**Authors:** Andreia-Alves Cardoso, Emerson-Tavares de Sousa, Carolina Steiner-Oliveira, Thaís-Manzano Parisotto, Marinês Nobre-dos-Santos

**Affiliations:** 1 Department of Pediatric Dentistry, Piracicaba Dental School, University of Campinas-UNICAMP, Limeira Avenue 901, Piracicaba-SP, Brazil; 2 Laboratory of Microbiology and Molecular Biology, Dental School, São Francisco University, Bragança Paulista – SP, Brazil

## Abstract

**Background:**

This research aimed to evaluate the salivary concentrations of fluoride (F-), calcium (Ca2+), and phosphate (Pi) after brackets bonding, and to identify the role of [F-], [Ca2+], and [Pi] on the development of active caries lesion (ACL) in individuals under fixed orthodontic treatment.

**Material and Methods:**

A longitudinal investigation with twenty-two individuals from 11 to 22 years of age was performed in four phases (baseline and after 1, 3, and 6 months). Analyses were carried out considering the salivary concentration of [F-], [Ca2+], and [Pi], as well as the caries index. Data were analyzed using the Friedman test, followed by the Wilcoxon test and the multivariate Cox model (*p*≤0.05).

**Results:**

1 and 3 months after appliance bonding, the [Ca2+] was statistically lower than after 6 months (*p*<0.0083). On the other hand, salivary [F-] and [Pi] did not show any significant difference during the follow-up. The Cox model demonstrated that the increase of 1 µg/mL in Ca2+ decreased the risk of ACL development by 27%. In conclusion, the levels of Ca2+ changed during orthodontic treatment.

**Conclusions:**

A high Ca2+ level in the saliva is a protective factor for ACL development over time.

** Key words:**Adolescents, bioinorganic chemistry, dental caries, orthodontic appliances.

## Introduction

The biofilm accumulation, facilitated by increased areas of stagnation, renders individuals submitted to orthodontic treatment more susceptible to developing active caries lesion (ACL) (1). Given this context, relevant modifications in the oral environment may enable the development of ACL, especially regarding saliva properties such as salivary flow rate and buffering capacity (2). Nevertheless, the influence of salivary electrolytes such as F-, Ca2+, and *Pi* on the development of ACL in individuals undergoing fixed orthodontic treatment must be explored.

In the oral cavity, salivary electrolytes play a relevant role in individual protection against dental caries. These electrolytes maintain saliva supersaturated with respect to hydroxyapatite and provide a positive influence in repairing tooth enamel. From a biochemical point of view, there is strong evidence regarding the physicochemical properties of saliva and its anticaries effect (3). However, limited evidence from clinical studies supports the protective effect of naturally-occurring salivary electrolytes (4).

Thus, this 6-month follow-up cohort study aimed 1) to evaluate the salivary concentrations of F-, Ca2+, and Pi before and after the placement of fixed orthodontic appliances, and 2) to identify the influence of F-, Ca2+, and Pi on the development of ACL in individuals under orthodontic treatment.

## Material and Methods

-Ethical Considerations

This study was approved by a Research Ethics Committee of a Brazilian University under the protocol No. 37395514.1.0000.5418. The guardians of participants under 18 years of age and all other individuals signed an informed consent form.

-Study Design and Sample

**Figure 1:**
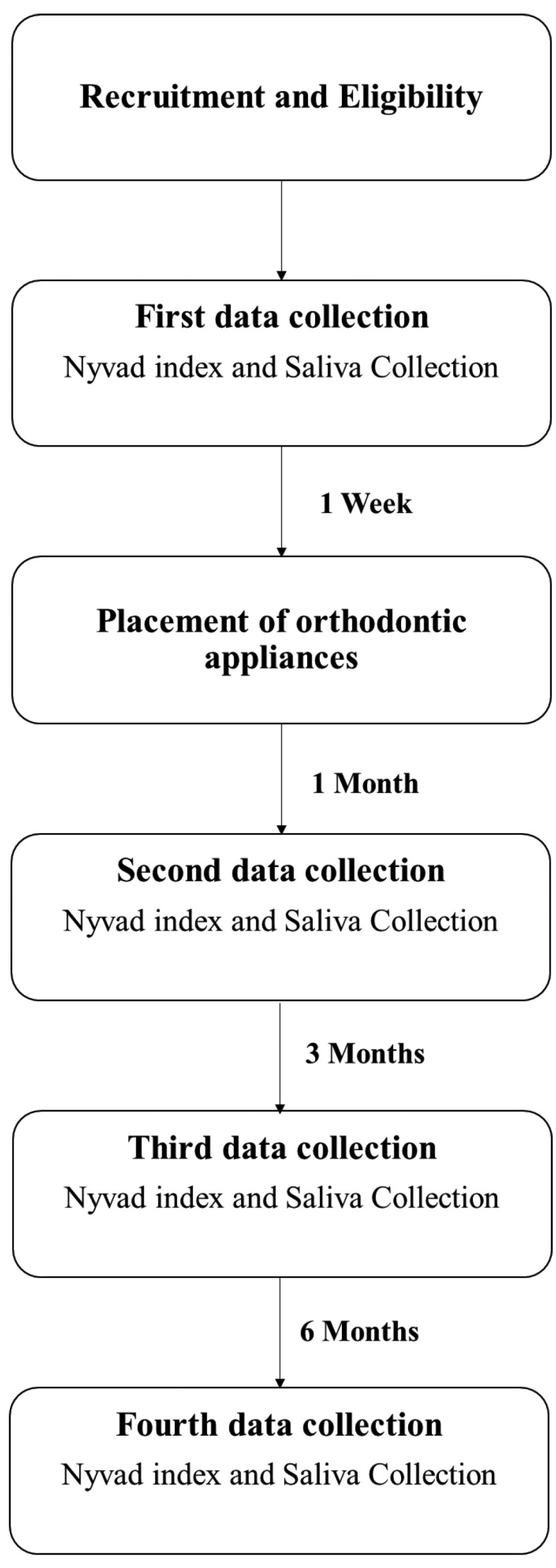
Experimental desing scheme of the study.

An experimental prospective cohort blind study was performed on a sample of 23 adolescents and young adults under fixed orthodontic treatment. Analyses were carried out in four distinct moments of collection: 1) One week before (baseline) the placement of orthodontic appliances, 2) 1 month after the placement of orthodontic appliances, 3) 3 months after the placement of orthodontic appliances, 4) 6 months after the placement of orthodontic appliances (Fig. 1).

The research protocol considered the clinical assessment of dental caries (Nyvad index) and saliva collection. Detailed methodological aspects of this cohort and information regarding the appliances system have been published elsewhere (2).

**Figure 2:**
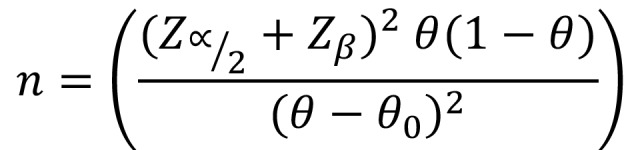
Formula.

The sample size was estimated to test whether there is a difference in the mean of white spot lesions between individuals undergoing orthodontic treatment and those who were not submitted to it (baseline). The sample size calculation considered the two-sided hypothesis and was performed following this formula (5), (Fig. 2):

Where, α was the probability of type I error (0.05), β was the probability of type II error (0.20), θ was the proportion of white spot lesion in exposed individuals (0.43) and θ0 was the proportions of white spot lesion in unexposed (0.13) individuals. The reference values to white spot lesions were based on Lucchese and Gherlone (6). The calculated sample size was 22. This study started with 23 volunteers to compensate for possible losses.

The participants were recruited according to priority from a waiting list for orthodontic treatment at the Pediatric Dentistry Department of the Piracicaba Dental School, University of Campinas ( Piracicaba, SP, Brazil). The inclusion criteria considered individuals of both sexes, aged from 11 to 22 years, with no primary teeth, with Angle Class I malocclusion, and absence of ACL, dental fluorosis, systemic diseases, dental hypoplasia, and severe dental crossing. The exclusion criteria were a history of antibiotic use in the 3 months prior to the beginning of the treatment, use of drugs that cause dry mouth for an extended amount of time, smoking, poor oral hygiene, periodontal disease, pregnancy, neuromotor disabilities, communication difficulties, and previous dental caries experience.

During every research protocol, the individuals were submitted to a comprehensive caries prophylactic program on a monthly basis. This program consisted of instructions (verbal and visual) regarding mechanical biofilm control, professional prophylaxis and daily use of the same fluoride dentifrice with 1.100 ppm of F. Besides this, the volunteers have access to 0.71 ppm of F in drinking water.

-Fixed Orthodontic Treatment 

For the orthodontic treatment, we used the Portia self-ligating ﬁxed orthodontic appliances system (Abzil, 3M, São José do Rio Preto, SP, Brazil), with Roth prescription and straight wire of 0.022″ x 0.028″ slot. The appliances were bonded using a light-cured Transbond XT adhesive (3M Unitek, Monrovia, California, USA), according to the manufacturer’s instructions. The brackets were applied to both arches on the buccal surface of incisors, canines, premolars and first permanent molars. The same orthodontist (first author) performed all treatment.

-Examiner Training and Dental Caries Assessment

Before the beginning of the experiment, a training exercise regarding the clinical examination of caries lesions was carried out in two steps (theoretical and clinical).

• Theoretical: Discussion of Nyvad index and analysis of clinical images.

• Clinical: The examiner and a gold standard (a researcher calibrated to the Nyvad index) performed clinical evaluations in a sample of 20 adolescents who were not included in the main sample. The Inter-rater reliability was assessed by the kappa test (k= 0.89) and the intra-rater reliability was checked one week after the first exam in 20% of the adolescents.

Clinical examinations were performed after the good reliability of training exercise. Individuals were examined at the dental office by a calibrated dentist (the first author), in a face-to-face position, using artificial light, a sterilized mouth mirror, and a WHO probe. A ball-ended dental probe explorer was used to remove debris and to enhance visualization and to conﬁrm questionable ﬁndings, checking loss of structure (cavitation) and surface texture (hard or rough/soft). Dental caries diagnosis was carried out according to Nyvad’s caries detection criteria, after the dental prophylaxis (7). This method consists of visual and tactile inspection of caries lesions, considering caries activity (active or inactive), extension and depth (intact, microcavities or cavitated surfaces) of the lesions, including all stages of the disease from white spot lesion to cavitation of dental surface. During the examination of each child, personal protective equipment as well as sterilized and individual clinical material were used.

-Saliva collection

For significant expression of electrolytes, stimulated saliva samples were collected in the morning, between 9:00 and 10:30 hours to avoid variations in the circadian rhythm, at least 1 hour after feeding. Participants were instructed to chew a piece of Parafilm® (Sigma Chemical Company, Chicago, Illinois, USA) and deposit the saliva in a Falcon® tube (BD Biosciences, Bedford, Massachusetts, USA) for 5 minutes. (Flow rate at baseline = 0.97 (0.50) mL/min, 1 month after orthodontic appliances placement = 1.17 (0.59) mL/min, 3 months after orthodontic appliances placement = 1.10 (0.53) mL/min, 6 months after orthodontic appliances placement = 1.06 (0.50) mL/min, ANOVA test with *p*-value = 0.66) Saliva samples were kept under refrigeration (2 to 8°C) in an ice container. The samples were centrifuged at 16097.2 g for 15 minutes and then stored at -40°C until analysis.

-Assessment of Saliva Inorganic Composition

Calcium and inorganic phosphate concentrations in saliva were analyzed by Inductively Coupled Plasma Optical Emission Spectrometry (ICP OES) (ICap 7400, Duo, Thermo Scientific). Fluoride concentration in centrifuged saliva samples was measured by the direct method, using an ion-selective electrode (8). The values obtained were expressed in µg/mL. Analyses were performed in duplicate.

-Statistical analysis

Statistical analysis was performed using SPSS software, version 21.0 (SPSS, Inc., Chicago, IL, USA). The dependent variables were F-, Ca2+, and Pi concentrations in saliva. The independent variable was the time of orthodontic treatment follow-up. Data were analyzed by the Friedman test, followed by the Wilcoxon test after non-Gaussian distribution. The level of significance established was 0.0083 for the two-tailed hypothesis, considering the Bonferroni adjustment of *p*-value to control the family-wise error rate. Multivariate Cox regression was carried out to determine the effect of risk factors (covariates: salivary F-, Ca2+, and Pi on ACL development. The Enter Method was employed to test covariates considering a *p*-value lower than 0.05.

## Results

At baseline, the caries experience of volunteers was 4.0 of restored surfaces (Interquartile Range: 8.0) without caries activity. One volunteer dropped out the study, thus, the final sample size was 22. The mean (standard deviation) age was 14 (2.9). The sex ratio was 0.83 male: 1.00 female volunteers. During the follow-up, it was observed that 59% of the sample (n=13) develop white spot lesions around the brackets after 3 months (40 dental surfaces) and 6 months (55 dental surfaces) from fixed orthodontic appliance placement. No cavity was identified in the follow-up period – Table 1.

**Table 1:**
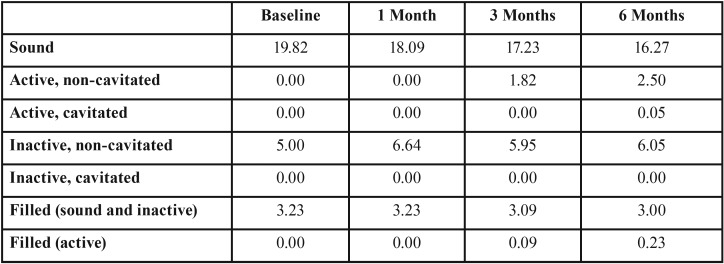
Number de dental surfaces during the follow-up periods according to Nyvad’s diagnostic criteria.

Table 2 shows that the Ca2+ concentrations 1 and 3 months after the beginning of the orthodontic treatment were statistically lower than after 6 months (*p*<0.0083). However, when the Ca2+ concentration was compared with the baseline values, no statistical difference at 1, 3, and 6 months could be evidenced (*p*>0.0083). Regarding salivary concentrations of F- and Pi, no significant change was demonstrated (*p*>0.05).

**Table 2:**
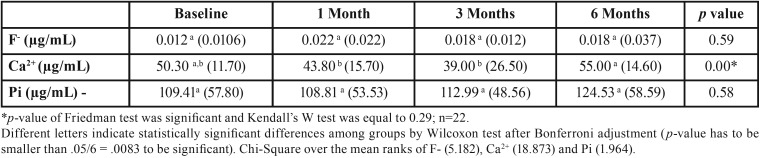
Medians and interquartile ranges of F-, Ca2+, and Pi concentrations in saliva at baseline and after the beginning of orthodontic treatment.

The Cox model demonstrated that an increase of 1 unit (µg/mL) of Ca2+ decreased the risk of ACL development by 27%. – Table 3.

**Table 3:**
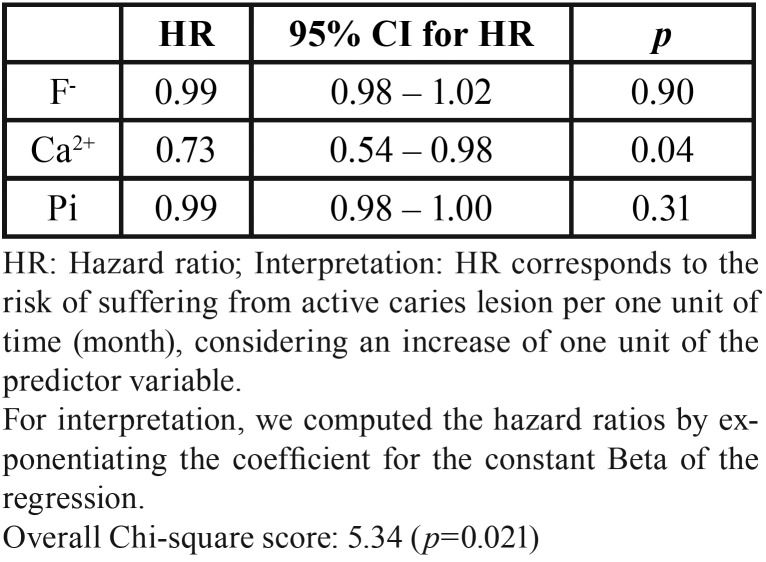
Cox’s regression of risk factors for the development of active caries.

## Discussion

In the oral cavity, calcium and phosphate ions are responsible for saliva supersaturation, cooperating to inhibit demineralization and enhance remineralization (9,10). Thus, salivary calcium and phosphate form a natural defense mechanism against dental caries (3,11). However, in the presence of a constant cariogenic challenge, it is possible that the frequent pH falls disrupt the equilibrium of these ions on the enamel surface (12,13). Regarding the relevance of these salivary ions on the dental caries development during the orthodontic treatment, our data demonstrated that the calcium concentration was lower after 1 and 3 months, whereas the inorganic phosphate concentration did not change during the follow-up period.

It is interesting to note that, as previously demonstrated by our group, the first clinical evidence of ACL was seen 3 months after the placement of orthodontic appliances (2). In line with these findings, this investigation pointed out that the deficiency in calcium concentration can be considered a risk factor (HR of 0.73) associated with ACL development in individuals submitted to orthodontic treatment. Thus, the results of this study emphasize a possible effect of the concentration of calcium ions on ACL development and its predictive value for caries activity in individuals under orthodontic treatment. In this context, it is hypothesized that the decrease in calcium concentration in saliva during the follow-up (1 and 3 months) may be a consequence of the demineralization and remineralization dynamic that is more pronounced in individuals at high risk of dental caries development and with frequent pH fluctuations during the day (14). More importantly, considering that the ion activity product (IAP) for hydroxyapatite is a function of the calcium concentration potentiated by 5 (IAP= (Ca2+)5 x (PO43-)3 x OH-) it is possible that a decrease in the calcium concentration may provide a deeper effect on the degree of saliva saturation in respect to hydroxyapatite and an increase in the critical pH for enamel dissolution, which would turn the enamel surface more susceptible to dental caries (4). In addition, there is also a possibility that salivary calcium may be complexed by acids, mainly lactate (15), reducing the saturation of saliva and increasing the driving force for demineralization, and consequently, the likelihood for the development of ACL.

In regards to fluoride salivary levels, although concentration increased during orthodontic treatment, no significant differences from baseline values were found. This can be considered a paradox, mainly because the salivary concentration of fluoride did not change enough to explain the development of ACL. It is well-known that available fluoride in saliva can be taken up into dental plaque, thus enhancing the process of teeth remineralization (16,17). However, our finding suggests that the severe cariogenic challenge around orthodontic appliances appears to require more fluoride than that delivered by the low fluoride concentrations in water and by the 1, 100-ppm fluoride dentifrice. In fact, it is evidenced that the use of professional methods of topical fluoride application, overcomes the preventive effects of the single-use of self-administrated (fluoride toothpaste) method in patients wearing orthodontic appliances; however, the level of evidence regarding the fluoride professional therapies against ACL is low (18). With this in mind, it must be pointed out that the diffusion and dissolution processes of this salivary ion on the tooth-pellicle-biofilm-saliva interface in individuals who do and who do not develop ACL should be investigated in detail considering the fluoride therapy.

The results of this investigation have clinical implications because they provide a broader comprehension concerning the behavior of salivary electrolytes over time in a sample of individuals under fixed orthodontic treatment. Besides this, this research highlights the placement of fixed orthodontic appliances as a high-risk situation for ACL development, as well as the limited effectiveness of the preventive effect of mechanical biofilm removal and daily use of fluoride dentifrice at 1.100 ppm and fluoridated water. However, to extrapolate these findings, more clinical studies are needed to determine the exact importance of salivary ions such as F-, Ca2+, and Pi on the physicochemical properties of saliva and, consequently, their influence on the development of ACL in individuals undergoing orthodontic treatment.

## Conclusions

Ca2+ levels changed during orthodontic treatment. These changes were more pronounced when the salivary Ca2+ composition at 6 months was compared with those at 1 and 3 months after the placement of orthodontic appliances. Conversely, no change could be noted regarding the salivary concentrations of F- and Pi.

The increasing of 1 µg/mL in Ca2+ decreased the risk of ACL development by 27%. Thus, an increased salivary concentration of Ca2+ may be considered a protective factor for ACL development over time in individuals undergoing orthodontic treatment.
